# The effects of acute exercise on episodic memory function among young university students: moderation considerations by biological sex

**DOI:** 10.15171/hpp.2019.14

**Published:** 2019-05-25

**Authors:** Lauren Johnson, Paul D. Loprinzi

**Affiliations:** Exercise & Memory Laboratory, Department of Health, Exercise Science and Recreation Management, The University of Mississippi, University, MS 38677, USA

**Keywords:** Cognition, Encoding, Learning, Memory, Physical activity

## Abstract

**Background:** The objective of this study was to evaluate potential sex-specific differences on episodic memory function and determine whether sex moderates the effects of acute exercise on episodic memory.

**Methods: ** A randomized controlled intervention was employed. This experiment was conducted among young University students (mean age = 21 years). Both males (n=20) and females (n=20)completed two counterbalanced laboratory visits, with one visit involving a 15-minute bout of moderate-intensity exercise prior to the memory task. The control visit engaged in a time matched seated task. Memory function (including short-term memory, learning, and long-term memory) was assessed from the RAVLT (Rey Auditory Verbal Learning Test).

**Results: ** We observed a significant main effect for time (P<0.001, ƞ^2^_p_= 0.77) and a marginally significant main effect for sex (P=0.06, ƞ^2^_p_= 0.09), but no time by sex by condition interaction(P=0.91, ƞ^2^_p_= 0.01). We also observed some suggestive evidence of a more beneficial effect of acute exercise on memory for females.

**Conclusion:** In conclusion, females outperformed males in verbal memory function. Additional research is needed to further evaluate whether sex moderates the effects of acute exercise on memory function.

## Introduction

Regular participation in physical activity has many health benefits, including a reduced risk of diabetes, cardiovascular disease and premature mortality,^1-6^ as well as enhancing psychological health.^7,8^ As such, from a health promotion perspective, promotion of physical activity is of critical importance for living a healthy lifestyle. In addition to the cardiometabolic benefits of physical activity, both acute and chronic physical activity participation demonstrate neurocognitive benefits.^9,10^ Promotion of physical activity is critical even among young adults, as cognitive decline may start to occur during the young adult years.^11^

Among young adults, experimental work from our laboratory has examined the effects of exercise on memory function, providing suggestive evidence that acute exercise may subserve episodic memory performance^12-18^ and may even attenuate a memory interference effect.^19^ We have previously detailed the dearth of research on this topic among this population.^20^ The mechanisms through which exercise may influence episodic memory function has also been extensively detailed by our group.^21-24^ Also, as we initially indicated elsewhere,^25^ very little research has evaluated the potential effects that biological sex may have on the relationship between acute exercise and memory function. Further, we recently detailed the literature on the role of sex on memory function, and how sex may moderate the effects of exercise on memory.^26^ However, to our knowledge, no study has specifically evaluated this potential exercise-induced sex-specific effect on episodic memory function.

As we have discussed elsewhere,^26^ in general, females outperform males on autobiographical memory (particularly with high retrieval support via verbal probing^27^), random word recall,^28^ story recall,^29^ auditory episodic memory,^30^ semantic memory (driven by superiority in fluency),^31^ and face recognition tasks.^32,33^ For race recognition tasks, females particularly have better recognition memory for female faces (and greater face perception).^34,35^ This may be a result of females being more familiar with female faces,^36^ which aligns with other work showing that recognition memory is superior for individuals who are of the same ethnic background as themselves.^37^ Females also have been shown to have greater scanning behavior at encoding,^33^ which may also contribute to their superior recognition memory. Females, however, do not outperform males for non-spatial memory, where males tend to perform better.^26^ In the context of exercise, there also appears to be a potential sex-specific effect on the response to exercise,^38^ which may subserve episodic memory function. For example, recent work suggests that older males (vs. older females) have a greater exercise-induced brain derived neurotrophic factor response.^39^

At this point, however, it is uncertain as to whether biological sex may moderate the effects of exercise on memory function. Thus, the purpose of this study was to twofold: 1) evaluate potential sex-specific differences on episodic memory function, and 2) evaluate whether biological sex moderates the effects of acute exercise on episodic memory function. We hypothesize that females (vs. males) will perform better on the non-exercise memory task, but there will be no differences in memory performance after acute exercise.

## Material and Methods

### Study design

A randomized controlled intervention was employed. This experiment was conducted among young University students in the authors’ laboratory. Data collection occurred between August and December of 2018. Both males and females completed two counterbalanced laboratory visits, with one visit involving a 15-minute bout of exercise prior to the memory task. The control visit engaged in a time-matched seated task. Each within-subject visit occurred around the same time of day (± 2 years) and occurred at least 24 hours after the first visit. Allocation concealment occurred by the lead researcher (L.J.) not looking at the allocation sheet until the participant arrived in the laboratory and completed the consent document. The random allocation sequence was generated by the principal investigator (P.L.). This study was approved by the authors’ institutional review board (#18-123) and participants provided written consent prior to participation.

### Participants and procedures

In total, 40 participants were recruited (20 males and 20 females). There were no drop outs after randomization. This was based on a conservative a-priori power analysis in G*Power (v. 3.1.9.2), using a RM-ANOVA within-between interaction model. With inputs of 0.05 (α), 0.80 (1-β), 2 groups (male/female), and 7 repeated measurements (trials), a sample size per group of 17 was needed to obtain sufficient statistical power. Our evaluated sample of 40 (n=20 per group) is in alignment with other related experiments.^14,16,40,41^ Recruitment occurred via a convenience-based, non-probability sampling approach (classroom announcement and word-of-mouth). Participants included undergraduate and graduate students between the ages of 18 and 35 years.

Additionally, and identical to other studies,^42^ participants were excluded if they:

- Self-reported as a daily smoker^43,44^

- Self-reported being pregnant^45^

- Exercised within 5 hours of testing^46^

- Consumed caffeine within 3 hours of testing^47^

- Had a concussion or head trauma within the past 30 days^48^

- Took marijuana or other illegal drugs within the past 30 days^49^

- Were considered a daily alcohol user (>30 drinks/month for women; >60 drinks/month for men)^50^

Table 1 displays the demographic and behavioral characteristics of the sample. Participants, on average, were 20.8 (0.9) years of age. The sample was equally distributed across sex, with 50% male and 50% female participants. There were no significant BMI (*P *= 0.98) or race-ethnicity (*P *= 0.11) differences across the two sexes. However, males (224 min/wk) were significantly (*P *= 0.006) more active than females (107.4 min/wk of moderate-to-vigorous physical activity).

### Exercise protocol

The exercise bout (a single exercise session) involved exercising on a treadmill for 15 minutes. Participants exercised at approximately 70% of their estimated heart rate max (220-age), which corresponds with moderate-intensity exercise.^51^

Immediately after the bout of exercise, participants rested in a seated position for 5 minutes. During this resting period, they played on-line game of Sudoku (described below) to prevent boredom. After this resting period, they commenced the memory assessment, as described below. We have experimental evidence that playing Sudoku does not prime or enhance memory function.

### Control protocol

For the control visit, and similar to other studies,^52^ participants completed a medium-level, on-line administered, Sudoku puzzle for 20-minutes. The website for this puzzle is located here: https://www.websudoku.com/.

### Memory assessment

Identical to our related experimental work,^14,16,41^ short-term and long-term memory (retrospective memory) were assessed using the standardized Rey Auditory Verbal Learning Test (RAVLT).^53^ Participants listened to and immediately recalled a recording of a list of 15 words (List A) five times in a row (Trials 1-5). Each word list (example words were: drum, curtain, bell, coffee, school, etc) was recorded at a rate of approximately 1 word per second. Participants then were asked to listen to and immediately recall a list of 15 new words (List B). Immediately following the recall of List B, participants were asked to recall the words from List A (Trial 6). Following Trial 6, participants watch a 20-minute video clip of “The Office – Bloopers”. After this 20-minute video clip, participants were asked to recall as many words as possible from List A (Trial 7).

### Additional assessments

Various demographic (e.g., BMI) and behavioral (i.e., habitual physical activity) assessments were completed to ensure that the groups were similar on these parameters. As a measure of habitual physical activity behavior, participants completed the Physical Activity Vital Signs Questionnaire to evaluate time spent per week in moderate-to-vigorous physical activity (MVPA).^54^ Height/weight (BMI; kg/m^2^) were measured to provide anthropometric characteristics of the sample. Lastly, before and at the end of the exercise and control conditions, heart rate (chest-strapped Polar monitor, F1 model) was assessed.

### Statistical analysis

All statistical analyses were computed in Jasp (v. 0.9.1). For the physiological data (heart rate), a 2 (sex) x 2 (exercise vs. control) x 2 (rest vs. endpoint) repeated measures ANOVA was computed. For the memory data, 2 (sex) x 2 (exercise vs. control) x 7 (trials) repeated measures ANOVA was computed. Statistical significance was set at an alpha of 0.05. Partial eta-square (η^2^_p_) was calculated as an estimate of effect size.

## Results

Table 2 displays the heart rate exercise responses. There was a significant main effect for time (F(1,38)=254.6, *P *< 0.001, ƞ^2^_p_=0.87), main effect for sex (F(1,38)=8.11, *P *= 0.007, ƞ^2^_p_=0.17), main effect for condition (F(1,38)=192.2, *P *< 0.001, ƞ^2^_p_=0.83), and time by condition interaction (F(1,38)=162.3, *P *< 0.001, ƞ^2^_p_=0.81). That is, heart rate was significant higher at the endpoint of exercise when compared to baseline, and heart rate was slightly higher for females (vs. males). Notably, there was no significant time by sex (F=0.35, *P *= 0.55, ƞ^2^_p_=0.01), sex by condition (F=0.62, *P *= 0.43, ƞ^2^_p_=0.01), or time by sex by condition (F=0.40, *P *= 0.52, ƞ^2^_p_=0.01) effects.

Table 3 displays the memory scores for both sexes and across the exercise and control periods. There was no main effect for condition (F(1,38)=0.66, *P *= 0.41, ƞ^2^_p_=0.02), but for females, more words were recalled for every trial during the exercise visit when compared to the control visit. We did not observe any interaction effects for time by sex (F(6,228)=0.76, *P *= 0.60, ƞ^2^_p_=0.02), time by condition (F(6,228)=0.53, *P *= 0.77, ƞ^2^_p_=0.01), sex by condition (F(1,228)=0.12, *P *= 0.72, ƞ^2^_p_=0.003), or time by sex by condition (F(6,228)=0.34, *P *= 0.91, ƞ^2^_p_=0.01). However, there was a significant main effect for time (F(6,228)=126.7, *P *< 0.001, ƞ^2^_p_=0.77) and a marginally significant main effect for sex (F(1,38)=3.76, *P *= 0.06, ƞ^2^_p_=0.09).

**Figure 1:**
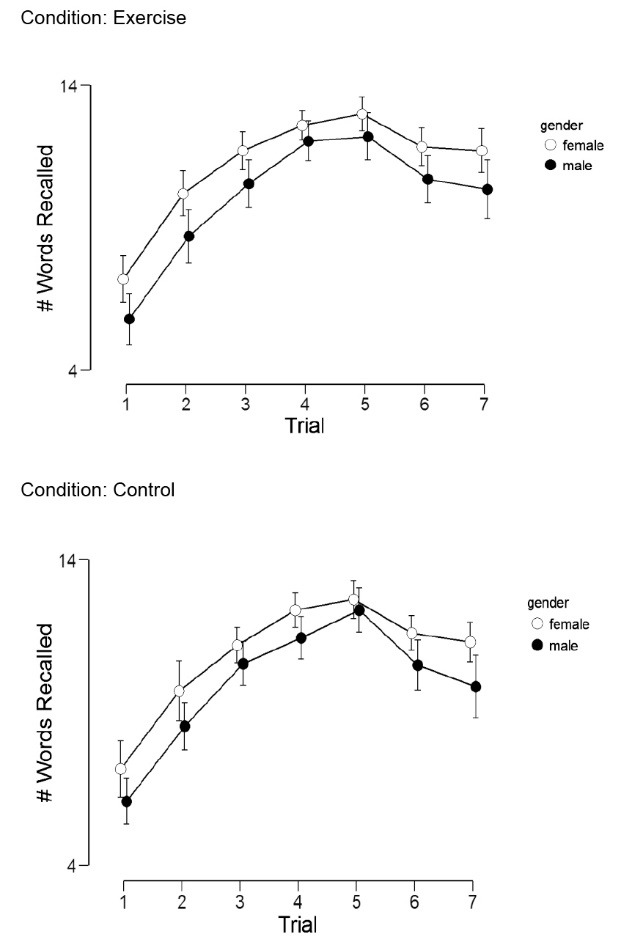
Memory scores across condition and sex. Error bars are 95% CI.

As expected, there was a significant main for time (F(6,228)=126.7, *P *< 0.001, ƞ^2^_p_=0.77). The number of recalled words increased over the first 5 trials, then decreased throughout the delayed assessment period. Regarding sex differences, there was a marginally significant main effect for sex (F(1,38)=3.76, *P *= 0.06, ƞ^2^_p_=0.09). As shown in Figure 1, for both the control and exercise visits, females (vs. males) had higher memory scores for every trial.

## Discussion

Previous experimental work suggests that acute exercise may subserve episodic memory function. Additionally, research demonstrates that females (vs. males) tend to outperform males on most non-spatial memory tasks. However, to our knowledge, no study has specifically evaluated whether there is a sex-specific exercise-related memory effect, which was the purpose of this experiment. The main findings of this experiment are as follows. Females outperformed males across all the 7 episodic memory trials (i.e., the word recall for the 7 trials), suggesting a sex-specific effect on short-term memory, learning, and long-term memory. Additionally, and although not statistically significant, females (but not males) had higher memory scores after exercising, when compared to their non-exercise visit.

As stated in the Introduction section, and as we have thoroughly addressed elsewhere,^26^ females tend to outperform males across nearly all non-spatial memory tasks. Our findings from the present experiment are in alignment with this body of literature. Our results also provide some suggestive evidence of an exercise-induced benefit for females; however, these results were not statistically significant, and thus, this should be interpreted with caution. These null exercise findings are likely not a result of a statistical power issue, as we were powered to observe such an effect. Further, our sample size is larger or similar to other studies that have observed statistically significant effects.^14,40^

Although other work has demonstrated that acute moderate-intensity physical activity is effective in enhancing memory function,^41^ our null exercise findings may, in part, be a result of the exercise intensity stimulus. Unlike moderate-intensity exercise, recent work demonstrates that higher-intensity exercise may be more effective in enhancing memory function.^55^ Our recent experimental work also supports episodic memory benefits from high-intensity exercise.^14^ Thus, future work should evaluate whether there are sex-specific, high-intensity exercise effects on memory function, and whether this occurs for spatial- and non-spatial memory function. In addition to exercise intensity, it is possible that our null exercise-induced effects may be a result of the time period in which long-term memory was assessed (i.e., 20-minute delay). Although research demonstrates that acute exercise can improve memory function when assessed at this 20-minute delay period,^14^ it is possible that a longer delay period may be needed for exercise-induced memory stabilization effects. Lastly, it is always important to be mindful that acute exercise may not always have beneficial effects on memory, which aligns with review work showing that, on average, only 48% to 71% of studies on exercise and memory observe a significant association.^20,56^

Limitations of this study include the homogenous sample of young, healthy adults. Thus, future work on this topic should consider evaluating other populations, including older adults with and without memory impairment.^57^ Strengths of this study include the experimental design and study novelty.

In conclusion, our experiment provides evidence that young adult females outperform males in verbal memory function. We did not observe sufficient evidence that sex moderates the effects of exercise on verbal memory performance, including short-term memory, learning, or long-term memory. Despite these findings, it is of critical importance to promote regular participation in physical activity, among all age populations, as habitual physical activity can help reduce a multitude of cardiovascular and cognitive morbidities.

## Ethical approval

This study was approved by the University of Mississippi’s ethics committee (#18-123).

## Competing interests

The authors declare that they have no competing interests.

## Funding

None.

## Authors’ contributions

LJ was involved in study conceptualization, data collection and manuscript revising; PL was involved in study conceptualization, statistical analyses, and manuscript writing.

**Table 1:** Demographic and behavioral characteristics of the sample

**Variable**	**Males**	**Females**	***P*** ** value**
N	20	20	
Age, mean years	20.95 (1.1)	20.65 (0.81)	0.34
Race, % non-Hispanic white	75.0	95.0	0.11
BMI, mean kg/m^2^	26.20 (3.4)	26.23 (4.8)	0.98
MVPA, mean min/wk	224.0 (161.0)	107.4 (72.1)	0.006

BMI, body mass index; MVPA, moderate-to-vigorous physical activity. Values in parentheses are standard deviations. Independent samples t-test was used to make comparisons across the continuous variables (e.g., age). A chi-square test was used to make comparisons across the categorical variables (e.g., race-ethnicity).

**Table 2:** Heart rate responses across the conditions

**Variable**	**Males**		**Females**		**Test-Statistic**
	**Exercise**	**Control**	**Exercise**	**Control**	
Baseline heart rate, mean bpm	84.55 (13.11)	72.65 (14.2)	93.75 (11.3)	83.4 (16.0)	F(time)=254.6, *P*<0.001, ƞ^2^_p_=0.87 F(sex)=8.11, *P*=0.007, ƞ^2^_p_=0.17 F(condition)=192.2, *P*<0.001, ƞ^2^_p_=0.83 F(time x sex)=0.35, *P*=0.55, ƞ^2^_p_=0.01 F(time x condition)=162.3, *P*<0.001, ƞ^2^_p_=0.81 F(sex x condition)=0.62, *P*=0.43, ƞ^2^_p_=0.01 F(time x sex x condition)=0.40, *P*=0.52, ƞ^2^_p_=0.01
Endpoint heart rate, mean bpm	130.6 (10.2)	76.25 (16.3)	136.0 (11.3)	87.25 (18.4)	

Bpm, beats per minute. Values in parentheses are standard deviations. For the physiological data (heart rate), a 2 (sex) x 2 (exercise vs. control) x 2 (rest vs. endpoint) repeated measures ANOVA was computed.

**Table 3:** Memory scores across the experimental conditions and by sex

**Variable**	**Males**		**Females**		**Test-Statistic**
	**Exercise**	**Control**	**Exercise**	**Control**	
Trial 1, mean # words	5.80 (1.7)	6.10 (1.6)	7.20 (2.0)	7.15 (2.0)	F(time)=126.7, P<0.001, ƞ^2^_p_=0.77 F(sex)=3.76, P=0.06, ƞ^2^_p_=0.09 F(condition)=0.66, P=0.41, ƞ^2^_p_=0.02 F(time x sex)=0.76, P=0.60, ƞ^2^_p_=0.02 F(time x condition)=0.53, P=0.77, ƞ^2^_p_=0.01 F(sex x condition)=0.12, P=0.72, ƞ^2^_p_=0.003 F(time x sex x condition)=0.34, P=0.91, ƞ^2^_p_=0.01
Trial 2, mean # words	8.70 (2.4)	8.55 (1.7)	10.20 (2.5)	9.70 (2.0)	
Trial 3, mean # words	10.55 (2.4)	10.60 (2.0)	11.70 (2.2)	11.20 (1.9)	
Trial 4, mean # words	12.05 (1.9)	11.45 (2.1)	12.60 (2.5)	12.35 (1.8)	
Trial 5, mean # words	12.20 (1.9)	12.35 (1.9)	13.00 (2.6)	12.70 (2.4)	
Trial 6, mean # words	10.70 (2.4)	10.55 (2.4)	11.85 (2.6)	11.60 (2.3)	
Trial 7, mean # words	10.35 (2.9)	9.85 (3.0)	11.70 (3.0)	11.30 (2.4)	

Values in parentheses are standard deviations Trials 1-6 are the free-recall assessments of List A. Trial 7 is the free-recall delayed assessment of List A, which occurred 20-minutes after Trial 6. For the memory data, 2 (sex) x 2 (exercise vs. control) x 7 (trials) repeated measures ANOVA was computed.
